# Supplementary material for: A neuronal mechanism underlying decision-making deficits during hyperdopaminergic states
Source: Nat Commun. 2018 Feb 21;9:731. doi: 10.1038/s41467-018-03087-1 (PMC5821846; doi:10.1038/s41467-018-03087-1)
Supplement: Supplementary file 1 — Supplementary Information [file 41467_2018_3087_MOESM1_ESM.pdf]

Supplementary figures

Supplementary Figure 1

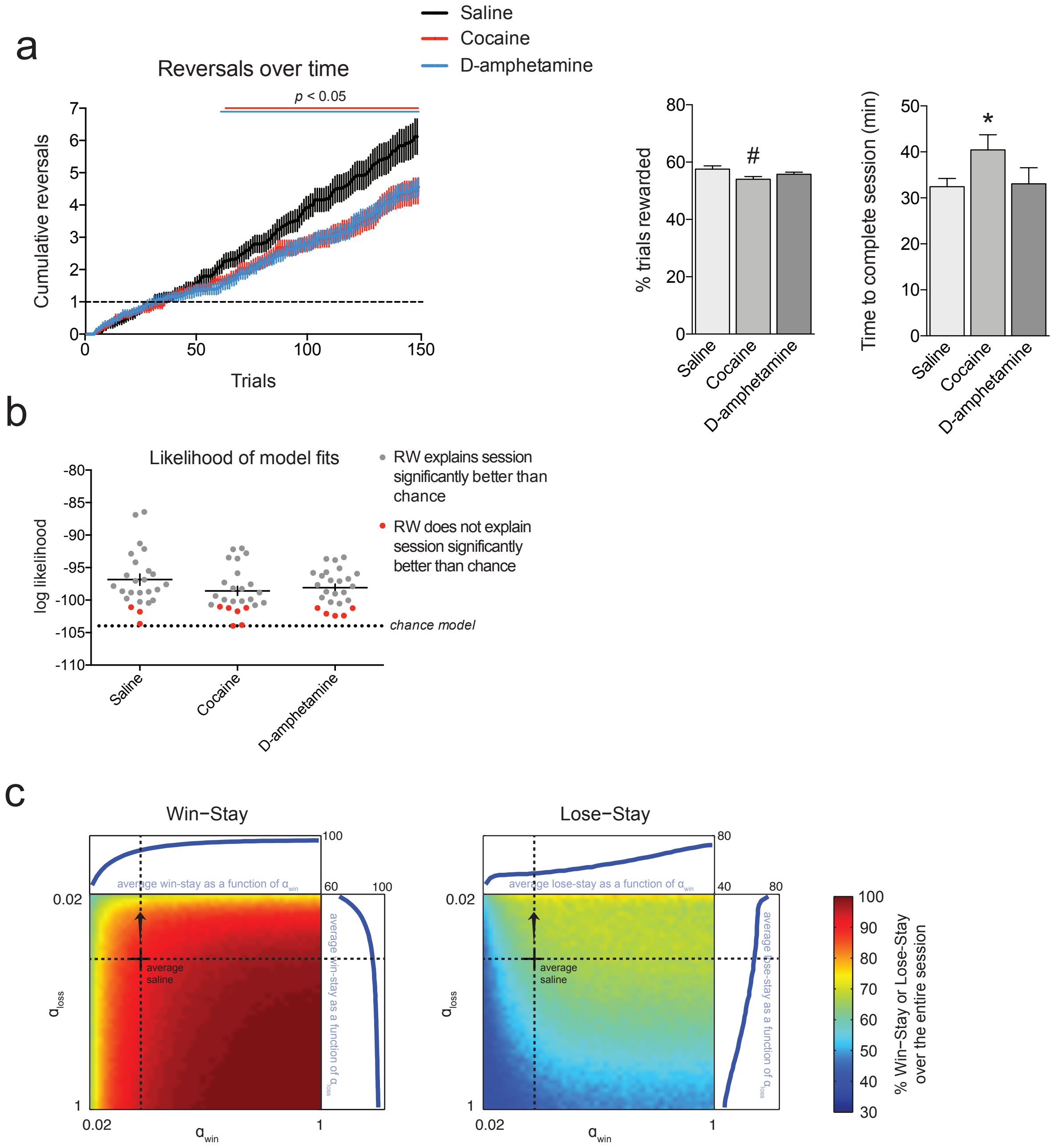

(**a**) Additional measures of the reversal learning task

(left panel) Plot of the cumulative reversals over time for all animals after systemic drug injection, confirming that the drug-induced performance impairment does not develop until after the first reversal (dashed line). Sidak's multiple comparisons test: $p < 0.05$ after trial 58 for D-amphetamine, $p < 0.05$ after trial 62 for cocaine.

(right panels)

Trials rewarded: one-way repeated measures ANOVA, $F(1.670, 40.08) = 3.998$, $p = 0.0327$. Post-hoc Sidak's test: cocaine vs saline, $t(24) = 2.358$, $p = 0.0530$; D-amphetamine vs saline, $t(25) = 1.561$, $p = 0.2461$.
Time to complete session: one-way repeated measures ANOVA, $F(1.930, 46.33) = 3.454$, $p = 0.0415$. Post-hoc Sidak's test: cocaine vs saline, $t(24) = 2.388$, $p = 0.0497$; D-amphetamine vs saline, $t(25) = 0.2042$, $p = 0.9744$.
Data shows mean ± standard error of the mean.
(**b**) Likelihood of model fits. Every dot represents an individual session. Cocaine and D-amphetamine did not significantly affect the fit of the model to the data (one-way repeated measures ANOVA, $F(2, 48) = 1.783$, $p = 0.1791$).
(**c**) Heatplot of simulated data showing how win- and lose-stay behavior (taken over the entire session) vary as a function of learning rates α_{win} and α_{loss}. Data shown are the average of 100 simulations of each $\alpha_{win}/\alpha_{loss}$ combination, with choice stochasticity factor β fixed at its mean for visualization purposes ($\beta = 6.7$). Dashed black lines show the average estimated learning rates after saline injection. The win-stay parameter is relatively stable for high learning rates compared to lose-stay, while lose-stay is more stable for lower learning rates. Hence, a decline of the average negative learning rate α_{loss} by ~2/3 more strongly affects win-stay than lose-stay behavior, providing an explanation for the observation that cocaine and D-amphetamine affect win-stay, but not lose-stay behavior. In contrast, when baseline learning rates would have been high, a decrease in α_{loss} would have resulted in an increase in lose-stay, without affecting win-stay behavior. Thus, how learning rates affect win- and lose-stay behavior is dynamic, and this strongly depends on the baseline estimates of α_{win}, α_{loss} and β.

Supplementary Figure 2

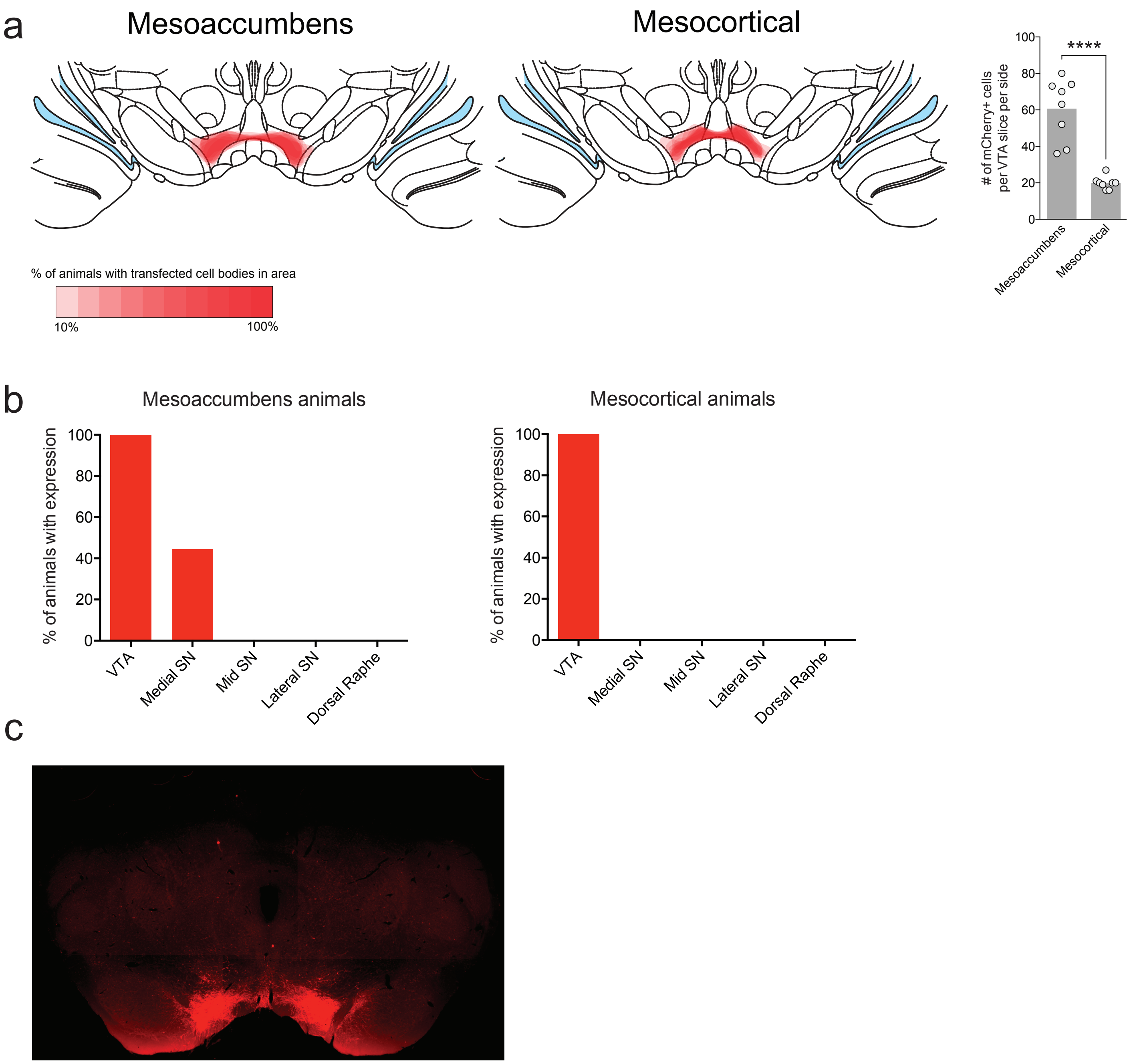

(**a**) (left) Spread of expression of Gq-mCherry in the midbrain. Shown is -5.40 mm posterior to Bregma. Atlas image adapted from Supplementary reference 1.
(right) Quantification of number of Gq-mCherry transfected neurons per group. Each dot represents a single animal. Significantly fewer neurons were transfected in the mesocortical group compared to the mesoaccumbens group (unpaired t-test, $t(14) = 6.713$, $p < 0.0001$).
(**b**) Quantification of expression of Gq-mCherry in the midbrain. In mesoaccumbens animals, virus sometimes spread to the medialmost part of the substantia nigra (SN), although this was always less than 5% of total transfected neurons.
(**c**) Example histology image of an animal from the mesoaccumbens group, showing strong expression of Gq-mCherry in the VTA and modest expression in the medial SN.

Supplementary Figure 3

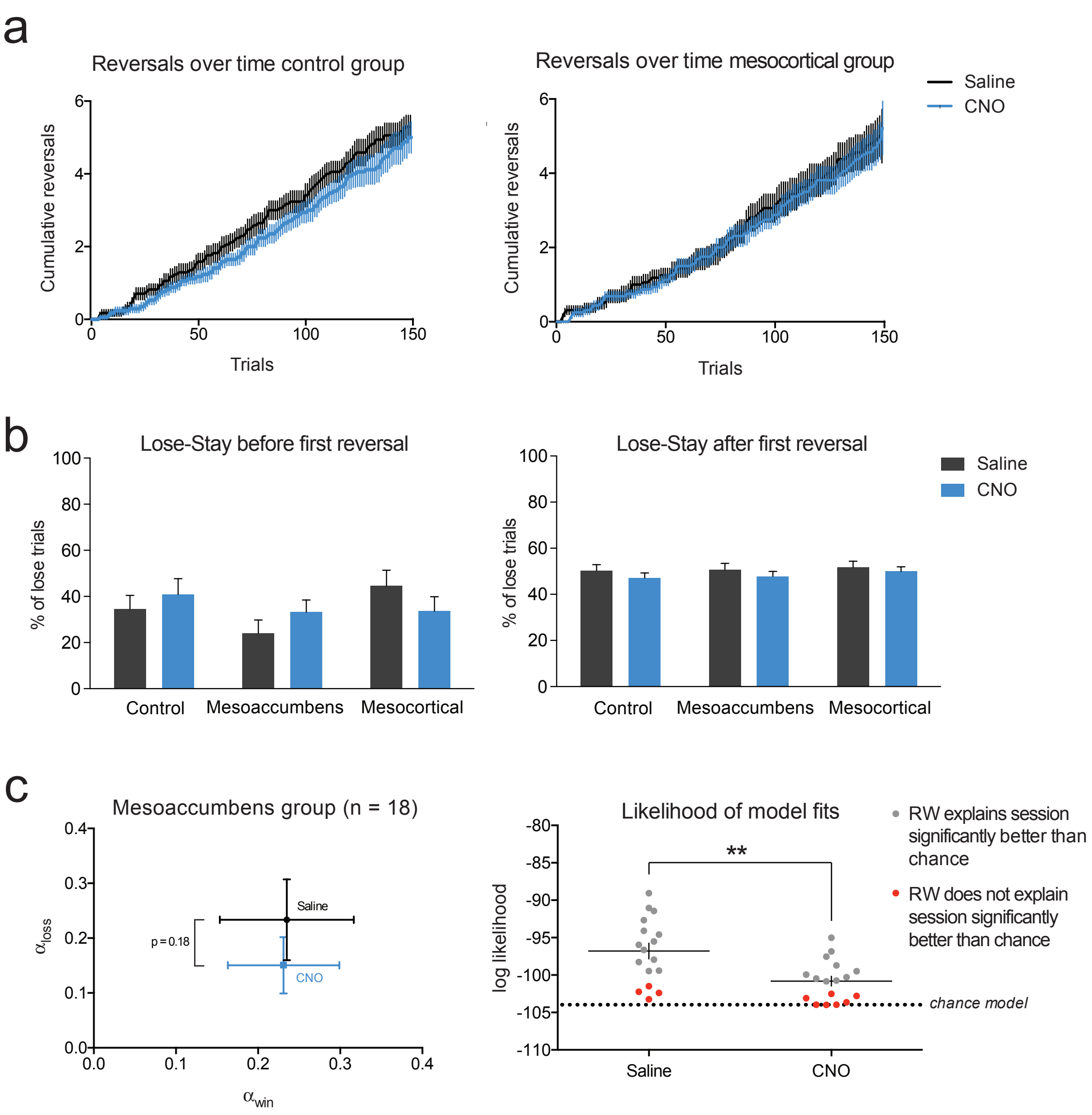

(**a**) No effect of CNO treatment on the cumulative reversals over time for the control group and the mesocortical group (two-way repeated measures ANOVA for control group: main effect of CNO, $F(1, 16) = 2.919$, $p = 0.1068$; trials × CNO interaction, $F(149, 2384) = 0.7633$, $p = 0.9838$; two-way repeated measures ANOVA for data mesocortical group: main effect of CNO, $F(1, 15) = 0.2858$, $p = 0.6007$; trials × CNO interaction, $F(148, 2220) = 0.5058$, $p > 0.9999$).

(**b**) Lose-stay behavior during reversal learning is not affected by DREADD stimulation of either pathway.

Left: two-way repeated measures ANOVA; main effect of CNO, $F(1, 40) = 0.1325$, $p = 0.7178$; group × CNO interaction, $F(2, 40) = 2.136$, $p = 0.1314$.

Right: two-way repeated measures ANOVA; main effect of CNO, $F(1, 50) = 1.392$, $p = 0.2436$; group × CNO interaction, $F(2, 50) = 0.045$, $p = 0.9556$.

(**c**) (left panel) Model fit on the reversal learning data of the mesoaccumbens group. DREADD activation altered α_{loss} in the same direction as cocaine and D-amphetamine, although not significantly so (one-tailed Wilcoxin matched-pairs signed rank test, $W = -41.00$, $p = 0.1764$).

(right panel) Mesoaccumbens activation resulted in a significantly poorer fit of the model to the data (paired t-test, $t(16) = 3.224$, $p = 0.0053$). This seems consistent with the observation that during mesoaccumbens hyperactivity, both win-stay (Fig. 2e) and lose-stay behavior (Supplementary Figure 3b) are around chance level (50%), making the Rescorla-Wagner model a suboptimal descriptor of the animals' behavior.

Supplementary Figure 4

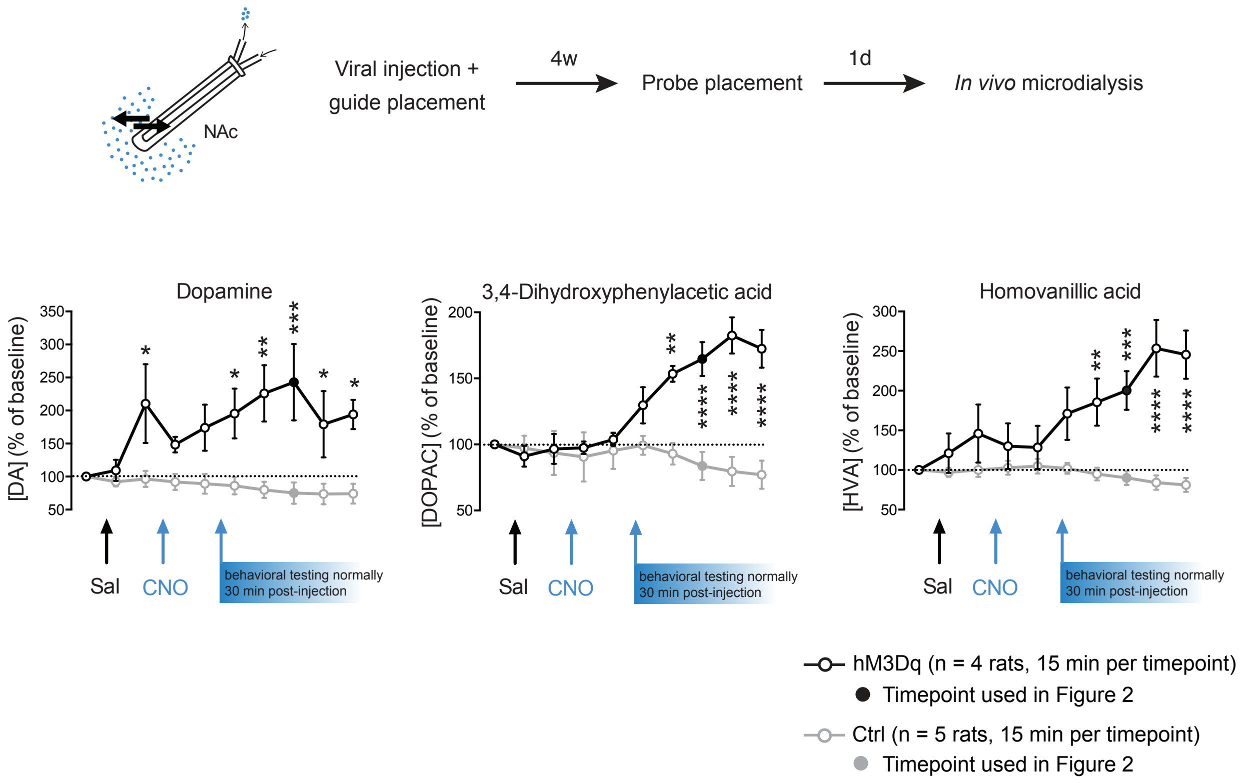

In vivo microdialysis performed in the NAc showed increased baseline levels of DA and its metabolites after activation of the mesoaccumbens pathway by CNO (n = 4 animals DREADD group, n = 5 animals control group)

Two-way repeated measures ANOVA, with factors treatment and timepoints:

DA:

Main effect of treatment: $F(1,7) = 11.83$, $p = 0.0108$

Treatment × Time interaction effect: $F(9,63) = 4.11$, $p = 0.0003$

DOPAC:

Main effect of treatment: $F(1,7) = 9.77$, $p = 0.0167$

Treatment × Time interaction effect: $F(9,63) = 15.69$, $p < 0.0001$

HVA:

Main effect of treatment: $F(1,7) = 9.01$, $p = 0.0199$

Treatment × Time interaction effect: $F(9,63) = 23.65$, $p < 0.0001$

Post-hoc LSD tests: * $p < 0.05$, ** $p < 0.01$, *** $p < 0.001$, **** $p < 0.0001$). Note a possible type I error at time point 3 in the DA graph.

Supplementary Figure 5

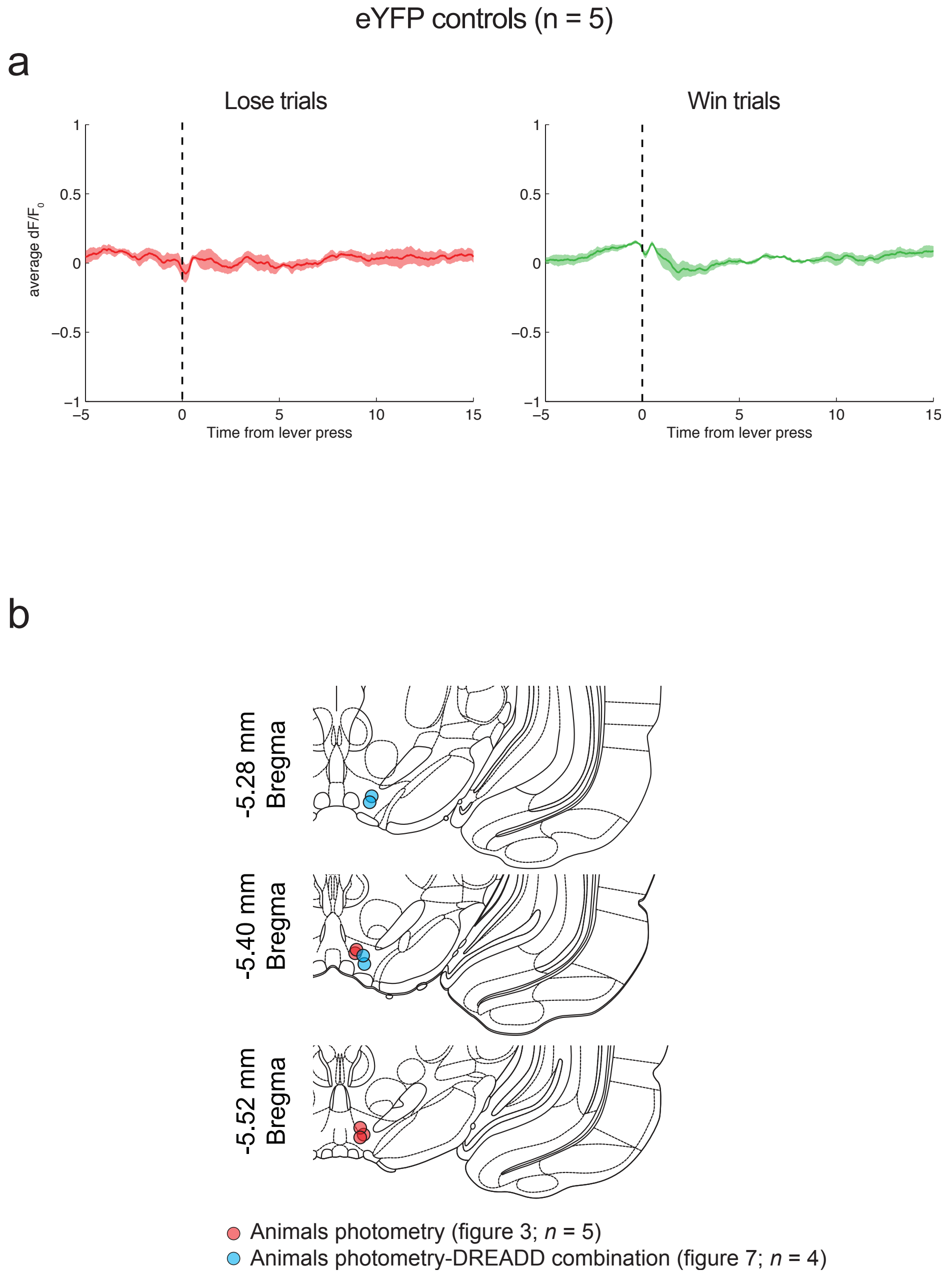

Fiber photometry
(**a**) Photometry responses during reversal learning in animals injected with the control fluorophore AAV-hSyn-eYFP (mean ± standard error of the mean).
(**b**) Fiber placement of animals used in photometry recordings. Atlas image adapted from Supplementary reference 1.

Supplementary Figure 6

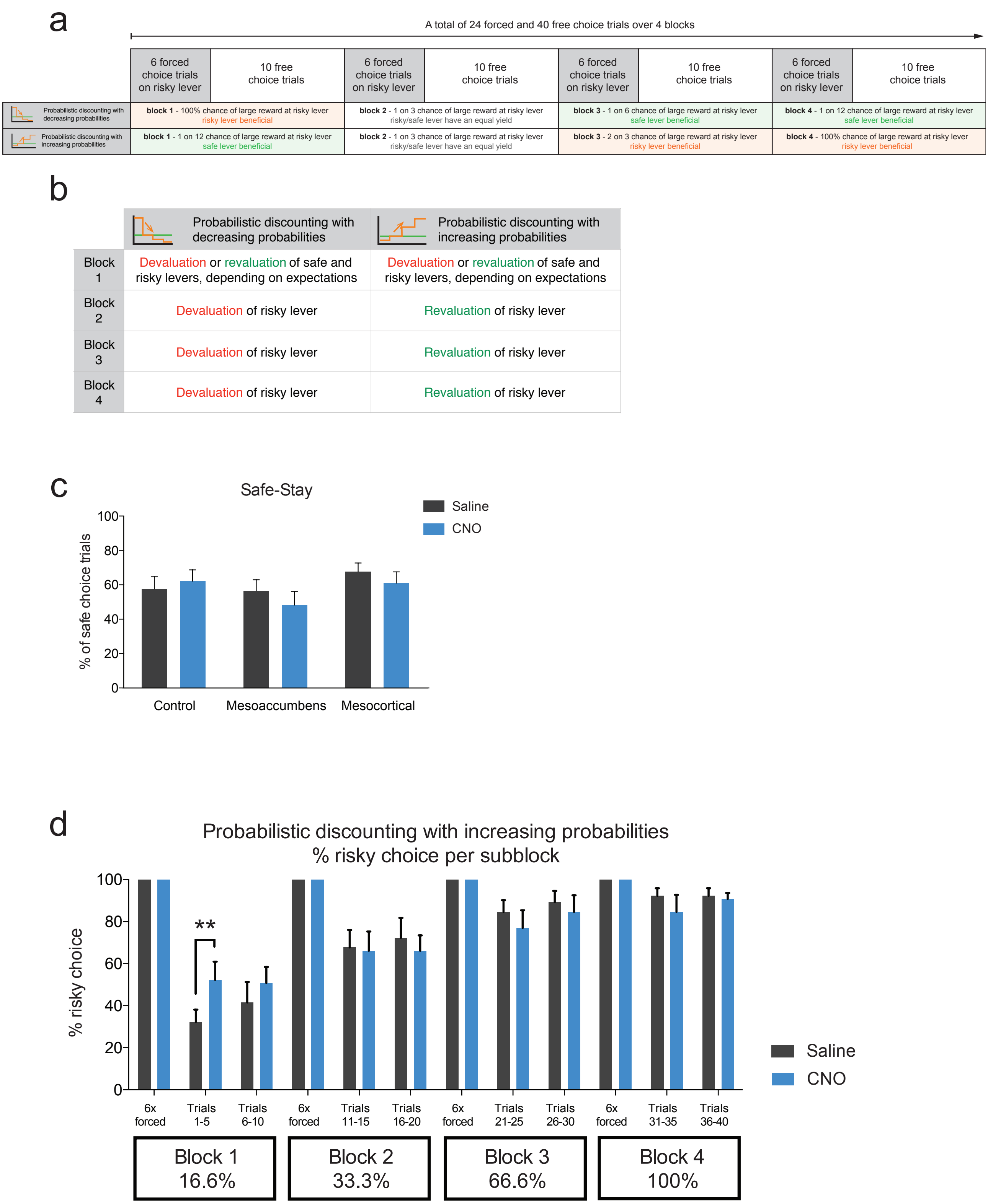

Probabilistic discounting task.

(**a**) In the probabilistic discounting task with decreasing probabilities across trial blocks, responding on the risky lever is economically beneficial in the first block, responding on the safe lever is beneficial in the last two blocks. In the second block, the yield of both levers is equal. The opposite is true for the version of the task with increasing probabilities across trial blocks.

(**b**) Depending on a priori knowledge, in the first block of the probabilistic discounting task, de- and revaluative mechanisms are needed to determine the reward value of the safe and risky levers. Assuming that a proper neuronal representation of lever value has been established at the end of the first block, subsequent blocks in the probabilistic discounting task with decreasing probabilities (left column) involve devaluative mechanisms, whereas the probabilistic discounting task with increasing probabilities (right column) involve revaluative mechanisms.
(**c**) Safe-stay behavior, defined as the percentage of safe choice trials followed by another safe choice, was unaffected by CNO treatment (two-way repeated measures ANOVA, main effect of CNO, $F(1, 34) = 1.050$, $p = 0.3127$; group × CNO interaction, $F(2, 34) = 1.365$, $p = 0.2690$).
(**d**) Percentage choice of the risky lever in the probabilistic discounting task with increasing probabilities during mesoaccumbens stimulation. Only in the first 5 trials of block 1, mesoaccumbens activation increased the choice for the risky lever, despite the low chance on reward (Fisher's LSD test in block 1: $t = 2.652$, $p = 0.0096$. In all other blocks: $p > 0.2$).

Supplementary Figure 7

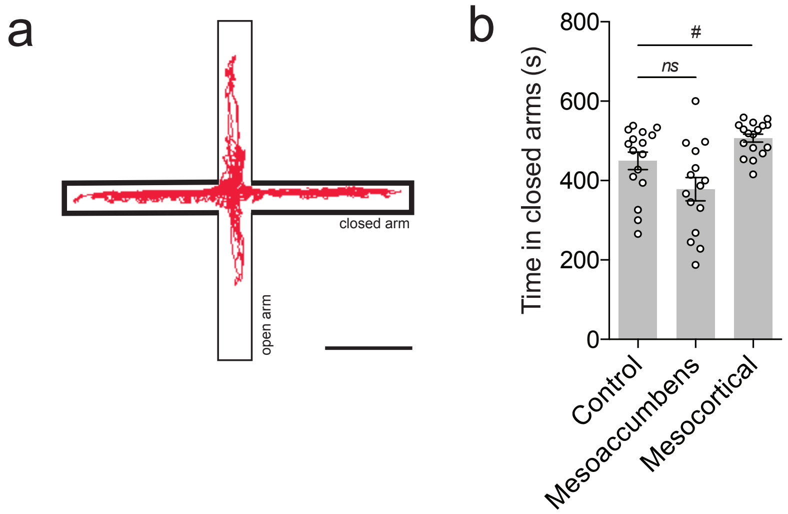

Elevated plus maze

(**a**) Example track of a control animal in the elevated plus maze. Red line indicates the track of the animal's center point. Scalebar, 25 cm.

(**b**) Total time spent in the closed arms of the elevated plus maze. Stimulation of the mesocortical pathway showed a trend towards increased anxiety, whereas stimulation of the mesoaccumbens pathway had no effect on behavior (unpaired t-test with Welch's correction for unequal variance, Bonferonni corrected for 2 comparisons; $F(26.22)_{uncorrected} = 1.943$, $p = 0.1256$ for mesoaccumbens versus control, $F(21.25)_{uncorrected} = 2.378$, $^{\#}p = 0.053$ for mesocortical versus control). n = 16 control, n = 15 mesoaccumbens, n = 17 mesocortical.

Supplementary Figure 8

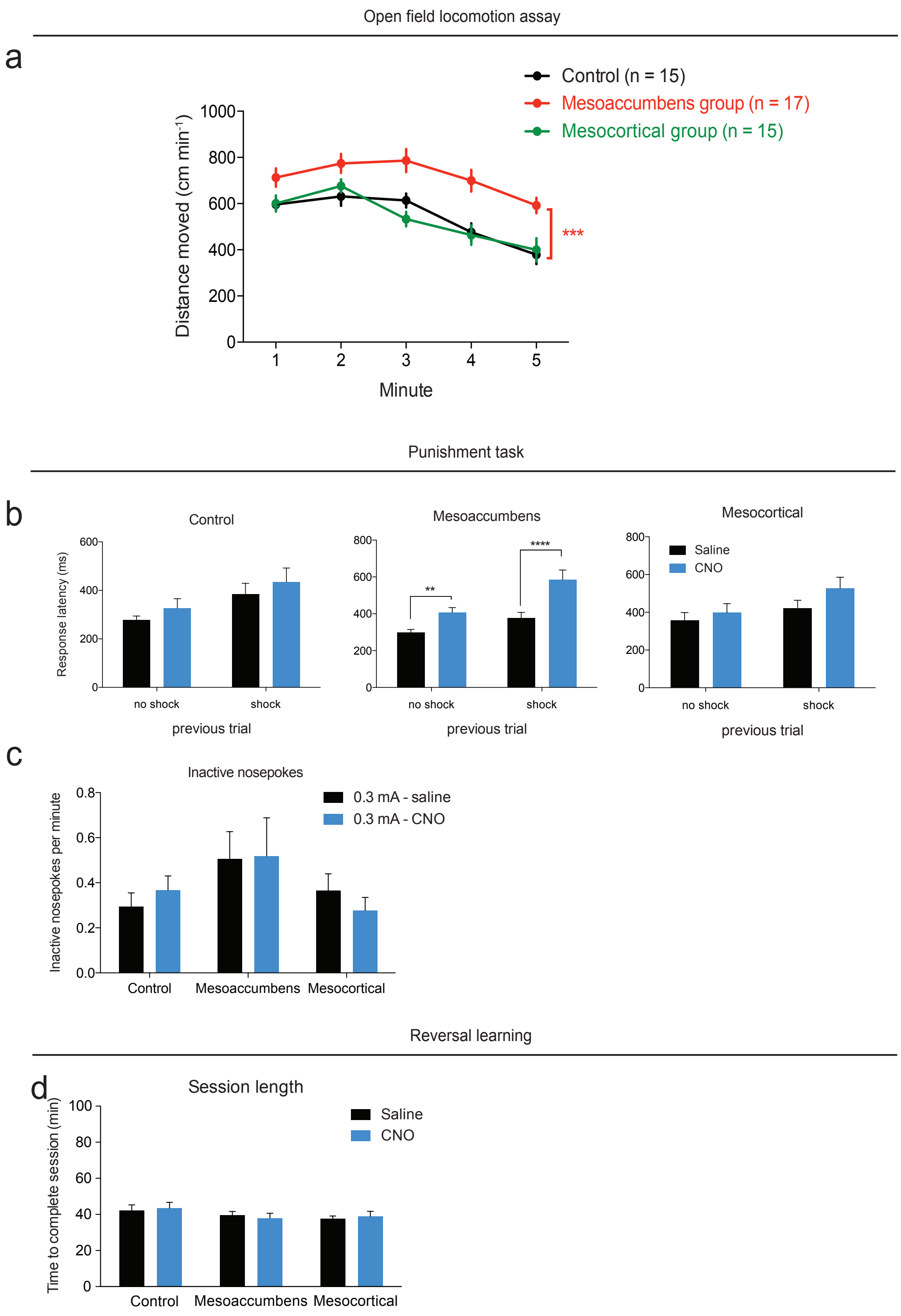

(*Figure legends on next page*)

(**a**) Mesoaccumbens stimulation increases locomotion (Sidak's multiple comparisons test, mesoaccumbens versus control, $t(44) = 4.383$, $p = 0.0001$; mesocortical versus control, $t(44) = 0.1096$, $p = 0.9925$). All animals received CNO.
(**b**) Reaction times in the punishment task (based on trials 11-30). Receiving a foot shock during a trial robustly increased the reaction time during the subsequent trial in all three groups (two-way repeated measures ANOVA; main effect of shock, all groups $p < 0.01$). In addition, a significant main effect of CNO ($F(1,9) = 20.97$, $p = 0.0013$) and a significant shock × CNO interaction ($F(1,9) = 8.271$, $p = 0.0183$) were observed in the mesoaccumbens group. Post-hoc Sidak's multiple comparisons test revealed a significant slowing of responding after mesoaccumbens activation after a no-shock trial ($t(9) = 4.532$, $p = 0.0028$), as well as after a shock trial ($t(9) = 8.599$, $p < 0.0001$).
(**c**) Mesocortical or mesoaccumbens activation did not affect inactive nose poking in the punishment task (2-way repeated measures ANOVA; main effect of CNO, $F(1,25) < 0.0001$, $p = 0.9946$; group × CNO interaction, $F(2, 25) = 0.3164$, $p = 0.7316$).
(**d**) Time animals needed to complete the 150 trials of the reversal learning session was unaffected by CNO treatment (two-way repeated measures ANOVA; main effect of CNO, $F(1, 47) = 0.0439$, $p = 0.8350$; group × CNO interaction, $F(2, 47) = 0.2961$, $p = 0.7451$).

Supplementary Figure 9

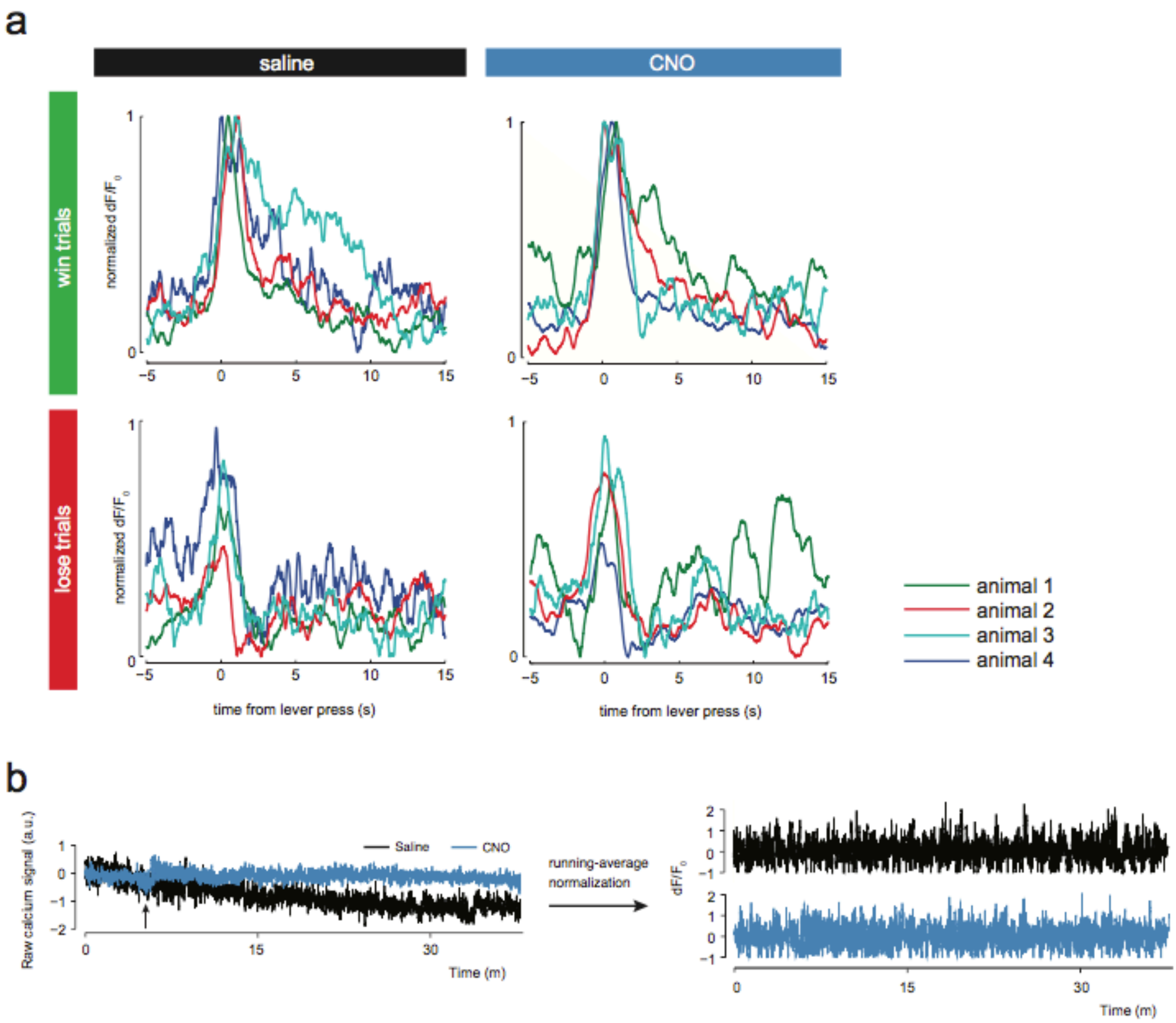

Photometry recordings of VTA DA neurons during DREADD activation.

(**a**) Data from individual animals from figure 7a.
(**b**) To correct for bleaching, raw calcium signal was converted to dF/F_0 values by normalizing to a running-average baseline.

Supplementary tables

Model	# of free parameters	Parameter estimates (mean ± SEM)			aggregate LL	significance model improvement
		α_{win}	α_{loss}	β		
M_0	0				-2599	
M_1	2	0.26 ± 0.05		2.0 ± 0.8	-2434	$M_1 > M_0$<br>$\chi^2(2) = 331.2$<br>p = 0
M_2	3	0.23 ± 0.06	0.31 ± 0.06	6.7 ± 1.7	-2421	$M_2 > M_1$<br>$\chi^2(1) = 24.9$<br>$p = 6.1 \times 10^{-7}$
	Constraints	[0 1]	[0 1]	[0 20]		

Supplementary table 1 Model fits, performed on baseline behavior (i.e., after saline treatment) in the reversal learning task in the *n* = 25 rats from figure 1. Model 1 (‘M_1’) is the classical Rescorla-Wagner model, whereas model 2 (‘M_2’) uses separate learning rates for reward (α_{win}) and punishment (α_{loss}) learning. Since the tested models are nested (M_1 is a special case of M_2), model comparison was performed using the likelihood-ratio test. M_0 is the baseline model, in which choice behavior is random (p = 0.5 for every trial).

	Parameter estimates		
	α_{win}	α_{loss}	β
	Learning from positive RPE	Learning from negative RPE	Choice stochasticity
Saline	0.23 ± 0.06	0.31 ± 0.06	6.7 ± 1.7
Cocaine	0.30 ± 0.07	0.13 ± 0.05 **	5.2 ± 1.4
D-amphetamine	0.26 ± 0.08	0.11 ± 0.02 *	8.8 ± 1.8

Supplementary table 2 Best-fit model parameters, estimated by maximizing the log likelihood for the model given the choice sequences in every session. Wilcoxon matched-pairs signed rank test with Bonferroni correction, α_{loss}: cocaine versus saline, *p* = 0.0046; D-amphetamine versus saline, *p* = 0.032. For additional statistics see the Methods checklist.

Supplementary table 3 Supplementary statistics

Fig	Test used	*n*	what?	p value	test statistic
1b, left	one-way RM anova w/ Geisser-Greenhouse correction	repeated measures in n = 25	wild-type Crl:WU rats	p = 0.5492	F(1.686, 40.47) = 0.5550
1b, right	one-way RM anova w/ Geisser-Greenhouse correction	repeated measures in n = 25	wild-type Crl:WU rats	p = 0.0037	F(1.793, 43.04) = 6.792
1b, right	post-hoc Sidak's test	repeated measures in n = 25	wild-type Crl:WU rats	coc vs sal:<br>p = 0.0102<br><br>amph vs sal:<br>p = 0.0197	coc vs sal:<br>t(24) = 3.081<br><br>amph vs sal:<br>t(24) = 2.801
1c	one-way RM anova w/ Geisser-Greenhouse correction	repeated measures in n = 25	wild-type Crl:WU rats	p = 0.4403	F(1.695, 40.67) = 0.7931
1d, 1st panel	one-way RM anova w/ Geisser-Greenhouse correction	repeated measures in n = 25	wild-type Crl:WU rats	p = 0.2125	F(1.624, 29.23) = 1.645
1d, 2nd panel	one-way RM anova w/ Geisser-Greenhouse correction	repeated measures in n = 25	wild-type Crl:WU rats	p = 0.7220	F(1.858, 44.60) = 0.3060
1d, 3rd panel	one-way RM anova w/ Geisser-Greenhouse correction	repeated measures in n = 25	wild-type Crl:WU rats	p = 0.6691	F(1.920, 46.08) = 0.3927
1d, 4th panel	one-way RM anova w/ Geisser-Greenhouse correction	repeated measures in n = 25	wild-type Crl:WU rats	p = 0.0007	F(1.741, 41.79) = 9.360
1d, 4th panel	post-hoc Sidak's test	repeated measures in n = 25	wild-type Crl:WU rats	coc vs sal:<br>p = 0.0009<br><br>amph vs sal:<br>p = 0.0336	coc vs sal:<br>t(24) = 4.042<br><br>amph vs sal:<br>t(24) = 2.567
1g	Wilcoxon matched-pairs signed rank test, Bonferroni corrected	repeated measures in n = 25	wild-type Crl:WU rats	ALPHA LOSS<br><br>coc vs sal:<br>p = 0.0023<br>corr p = 0.0046<br>amph vs sal:<br>p = 0.0160<br>corr: p = 0.032<br><br>ALPHA WIN<br><br>coc vs sal:<br>p = 0.4223<br>corr p = 0.8446<br>amph vs sal:<br>p = 0.9643<br>corr p > 0.999<br><br>BETA<br>(not shown)<br><br>coc vs sal:<br>p = 0.4578<br>corr p = 0.9156<br>amph vs sal:<br>p = 0.6150<br>corr p > 0.999	ALPHA LOSS<br><br>coc vs sal:<br>W = -219.0<br>amph vs sal:<br>W = -177.0<br><br>ALPHA WIN<br><br>coc vs sal:<br>W = 58.00<br>amph vs sal:<br>W = 4.000<br><br>BETA<br>(not shown)<br><br>coc vs sal:<br>W = -57.00<br>amph vs sal:<br>W = 39.00
1h, 1st panel	one-way RM anova w/ Geisser-Greenhouse correction	repeated measures in n = 25	simulated rats (average of 5 simulations per rat)	p = 0.8619	F(1.505, 36.12) = 0.0897
1h, 2nd panel	one-way RM anova w/ Geisser-Greenhouse correction	repeated measures in n = 25	simulated rats (average of 5 simulations per rat)	p = 0.0114	F(1.727, 41.45) = 5.335

Fig	Test used	*n*	what?	p value	test statistic
1h, 2nd panel	post-hoc Sidak's test	repeated measures in n = 25	simulated rats (average of 5 simulations per rat)	coc vs sal:<br>p = 0.0411<br><br>amph vs sal:<br>p = 0.0215	coc vs sal:<br>t(24) = 2.475<br><br>amph vs sal:<br>t(24) = 2.764
1h, 3rd panel	one-way RM anova w/ Geisser-Greenhouse correction	repeated measures in n = 25	simulated rats (average of 5 simulations per rat)	p = 0.0090	F(1.885, 45.24) = 5.384
1h, 3rd panel	post-hoc Sidak's test	repeated measures in n = 25	simulated rats (average of 5 simulations per rat)	coc vs sal:<br>p = 0.0181<br><br>amph vs sal:<br>p = 0.0462	coc vs sal:<br>t(24) = 2.839<br><br>amph vs sal:<br>t(24) = 2.421
2b	none	mesoaccumbens<br>n = 9<br><br>mesocortical n = 8	wild-type Crl:WU rats	-	-
2c, left panel	two-way RM anova	Ctrl<br>n = 17<br>Mesoacc n = 17<br>Mesocort<br>n = 17	wild-type Crl:WU rats	CNO<br>p = 0.5409<br><br>CNO × group<br>p = 0.8968	CNO<br>F(1, 47) = 0.3794<br><br>CNO × group<br>F(2, 47) = 0.1092
2c, right panel	two-way RM anova	Ctrl<br>n = 17<br>Mesoacc n = 17<br>Mesocort<br>n = 17	wild-type Crl:WU rats	CNO<br>p = 0.0025<br><br>CNO × group<br>p = 0.0067	CNO<br>F(1, 47) = 10.22<br><br>CNO × group<br>F(2, 47) = 5.582
2c, right panel	post-hoc Sidak's test	Ctrl<br>n = 17<br>Mesoacc n = 17<br>Mesocort<br>n = 17	wild-type Crl:WU rats	Ctrl<br>p = 0.8667<br><br>Mesoacc<br>p < 0.0001<br><br>Mesocort<br>p = 0.9886	Ctrl<br>t(47) = 0.6971<br><br>Mesoacc<br>t(47) = 4.601<br><br>Mesocort<br>t(47) = 0.2874
2d	Sidak's test	Ctrl<br>n = 17<br>Mesoacc n = 17<br>Mesocort<br>n = 17	wild-type Crl:WU rats	p < 0.05 after trial 85	-
2e, left panel<br><br>2e, right panel	two-way RM anova	Ctrl<br>n = 17<br>Mesoacc n = 17<br>Mesocort<br>n = 17	wild-type Crl:WU rats	CNO<br>p = 0.7754<br><br>CNO × group<br>p = 0.9093<br><br>CNO<br>p = 0.0040<br><br>CNO × group<br>p = 0.0026	CNO<br>F(1, 50) = 0.0823<br><br>CNO × group<br>F(2, 50) = 0.0952<br><br>CNO<br>F(1, 50) = 9.07<br><br>CNO × group<br>F(2, 50) = 6.710
2e, right panel	post-hoc Sidak's test	Ctrl<br>n = 17<br>Mesoacc n = 17<br>Mesocort<br>n = 17	wild-type Crl:WU rats	Ctrl<br>p = 0.9647<br><br>Mesoacc<br>p < 0.0001<br><br>Mesocort<br>p = 0.9997	Ctrl<br>t(50) = 0.4258<br><br>Mesoacc<br>t(50) = 4.753<br><br>Mesocort<br>t(50) = 0.0836

Fig	Test used	*n*	what?	p value	test statistic
2f	two-way RM anova	Ctrl<br>n = 17<br>Mesoacc n = 17<br>Mesocort<br>n = 17	wild-type Crl:WU rats	CNO<br>p = 0.5491<br><br>CNO × group<br>p = 0.4293	CNO<br>F(1, 50) = 0.3638<br><br>CNO × group<br>F(2, 50) = 0.8601
2h, DA	two-way RM anova<br>(see Supplementary Figure 4)	n = 4 hM3Dq<br>n = 5 ctrl	wild-type Crl:WU rats	treatment group<br>p = 0.0108<br><br>treatment group × time point<br>p = 0.0003	treatment group<br>F(1,7) = 11.83<br><br>treatment group × time point<br>F(9,63) = 4.11
2h, DA	post-hoc test on timepoint 8	n = 4 hM3Dq<br>n = 5 ctrl	wild-type Crl:WU rats	t(70) = 4.574	p = 0.0002
2h, DOPAC	two-way RM anova<br>(see Supplementary Figure 4)	n = 4 hM3Dq<br>n = 5 ctrl	wild-type Crl:WU rats	treatment group<br>p = 0.0167<br><br>treatment group × time point<br>p < 0.0001	treatment group<br>F(1,7) = 9.77<br><br>treatment group × time point<br>F(9,63) = 15.69
2h, DOPAC	post-hoc test on timepoint 8	n = 4 hM3Dq<br>n = 5 ctrl	wild-type Crl:WU rats	t(70) = 5.081	p < 0.0001
2h, HVA	two-way RM anova<br>(see Supplementary Figure 4	n = 4 hM3Dq<br>n = 5 ctrl	wild-type Crl:WU rats	treatment group<br>p = 0.0199<br><br>treatment group × time point<br>p < 0.0001	treatment group<br>F(1,7) = 0.0199<br><br>treatment group × time point<br>F(9,63) = 23.65
2h, HVA	post-hoc test on timepoint 8	n = 4 hM3Dq<br>n = 5 ctrl	wild-type Crl:WU rats	t(70) = 4.111	p = 0.0008
2j, left panel	one-way RM anova<br><br>followed by post-hoc Holm-Sidak's test	n = 7	wild-type Crl:WU rats	1-way ANOVA:<br>p = 0.0024<br><br>post-hoc tests:<br>Sal/Sal v Sal/Flup<br>p = 0.5500<br><br>Sal/Sal v CNO/Sal<br>p = 0.0019<br><br>Sal/Sal v CNO/Flup<br>p = 0.2692<br><br>CNO/Sal v CNO/Flup<br>p = 0.0397	1-way ANOVA:<br>F(3, 18) = 7.098<br><br>post-hoc tests:<br>Sal/Sal v Sal/Flup<br>t(18) = 0.6092<br><br>Sal/Sal v CNO/Sal<br>t(18) = 4.264<br><br>Sal/Sal v CNO/Flup<br>t(18) = 1.523<br><br>CNO/Sal v CNO/Flup<br>t(18) = 2.741
2j, right panel	two-way RM anova followed by post-hoc Sidak's test	n = 7	wild-type Crl:WU rats	ANOVA:<br><br>Trials<br>p < 0.0001<br><br>Treatment<br>p = 0.1085<br><br>treatment × trials<br>p < 0.0001<br><br>post-hoc Sidak's test, CNO/Sal vs Sal/Sal p < 0.05 after trial 107<br><br>other treatments not significant in post-hoc vs Sal/Sal	Trials<br>F(149, 894) = 521.0<br><br>Treatment<br>F(3,18) = 2.332<br><br>treatment × trials<br>F(447,2682) = 2.130
3b, inset	unpaired t-test	win:<br>n = 81<br><br>lose:<br>n = 68	trials	p < 0.0001	t(147) = 5.446

Fig	Test used	*n*	what?	p value	test statistic
3c, mid	paired t-test<br>win vs lose	repeated measures in n = 5	TH::Cre rats	p = 0.0015	t(4) = 7.809
3c, right	paired t-test<br>before vs after (lose)<br><br>paired t-test<br>before vs after (win)	repeated measures in n = 5	TH::Cre rats	p = 0.0062<br><br>p = 0.3658	t(4) = 5.282<br><br>t(4) = 1.019
4b, left	Sidak's test	n = 12<br>ctrl	wild-type Crl:WU rats	block 1<br>p > 0.9999<br><br>block 2<br>p = 0.8916<br><br>block 3<br>p = 0.9907<br><br>block 4<br>p = 0.3911	block1<br>t(33) = 0.1007<br><br>block2<br>t(33) = 0.8056<br><br>block3<br>t(33) = 0.4028<br><br>block4<br>t(33) =1.611
4b,<br>mid	Sidak's test	n = 13<br>mesoacc	wild-type Crl:WU rats	block1<br>p = 0.0468<br><br>block 2<br>p = 0.8871<br><br>block 3<br>p = 0.4405<br><br>block 4<br>p = 0.0468	block1<br>t(36) = 2.649<br><br>block2<br>t(36) = 0.8152<br><br>block3<br>t(36) = 1.529<br><br>block4<br>t(36) = 2.649
4b, right	Sidak's test	n = 13<br>mesocort	wild-type Crl:WU rats	block 1<br>p = 0.9745<br><br>block 2<br>p = 0.0247<br><br>block 3<br>p = 0.4898<br><br>block 4<br>p = 0.8336	block1<br>t(36) = 0.5284<br><br>block2<br>t(36) = 2.906<br><br>block3<br>t(36) = 1.453<br><br>block4<br>t(36) = 0.9247
4b, insets	Sidak's test	ctrl<br>n = 12<br><br>mesoacc<br>n = 13<br><br>mesocort<br>n = 13	wild-type Crl:WU rats	ctrl<br>p = 0.7551<br><br>mesoacc<br>p = 0.0002<br><br>mesocort<br>p = 0.9510	ctrl<br>t(35) = 0.8999<br><br>mesoacc<br>t(35) = 4.467<br><br>mesocort<br>t(35) = 0.4803
4c	two-way RM anova	ctrl<br>n = 12<br><br>mesoacc<br>n = 13<br><br>mesocort<br>n = 13	wild-type Crl:WU rats	CNO<br>p = 0.0331<br><br>CNO ╳ group<br>p = 0.0016	CNO<br>F(1, 35) = 4.922<br><br>CNO ╳ group<br>F(2, 35) = 7.819
4c	Sidak's test	ctrl<br>n = 12<br><br>mesoacc<br>n = 13<br><br>mesocort<br>n = 13	wild-type Crl:WU rats	ctrl<br>p = 0.5082<br><br>mesoacc<br>p = 0.0004<br><br>mesocort<br>p = 0.7533	ctrl<br>t(35) = 1.275<br><br>mesoacc<br>t(35) = 4.320<br><br>mesocort<br>t(35) = 0.9027

Fig	Test used	*n*	what?	p value	test statistic
4d, left	two-way RM anova	ctrl<br>n = 12<br><br>mesoacc<br>n = 13<br><br>mesocort<br>n = 13	wild-type Crl:WU rats	CNO<br>p = 0.3620<br><br>CNO ╳ group<br>p = 0.2649	CNO<br>F(1, 34) = 0.8536<br><br>CNO ╳ group<br>F(2, 34) = 1.382
4d, right	two-way RM anova	ctrl<br>n = 12<br><br>mesoacc<br>n = 13<br><br>mesocort<br>n = 13	wild-type Crl:WU rats	CNO<br>p = 0.0026<br><br>CNO ╳ group<br>p = 0.0622	CNO<br>F(1, 34) = 10.61<br><br>CNO ╳ group<br>F(2, 34) = 3.017
4d, right	Sidak's test	ctrl<br>n = 12<br><br>mesoacc<br>n = 13<br><br>mesocort<br>n = 13	wild-type Crl:WU rats	ctrl<br>p = 0.9988<br><br>mesoacc<br>p = 0.0177<br><br>mesocort<br>p = 0.0203	ctrl<br>t(34) = 0.1358<br><br>mesoacc<br>t(34) = 2.936<br><br>mesocort<br>t(34) = 2.882
4f	Sidak's test	n = 8	wild-type Crl:WU rats	block 1<br>p = 0.1634<br><br>block 2<br>p = 0.9705<br><br>block 3<br>p = 0.8564<br><br>block 4<br>p = 0.9439	block1<br>t(36) = 2.091<br><br>block2<br>t(36) = 0.5503<br><br>block3<br>t(36) = 0.8805<br><br>block4<br>t(36) = 0.6604
4g	paired t-tests (uncorr.)	n = 8	wild-type Crl:WU rats	Performance<br>p = 0.0143<br><br>Win-Stay<br>p = 0.3236<br><br>Lose-Stay<br>p = 0.8491	Performance<br>t(12) = 2.862<br><br>Win-Stay<br>t(12) = 1.029<br><br>Lose-Stay<br>t(12) = 0.1944
5a, left panel	two-way RM anova	n = 9 ctrl<br>n = 8 mesoacc<br>n = 9 mesocort	wild-type Crl:WU rats	CNO<br>p = 0.0355<br><br>CNO ╳ group<br>p = 0.5001	CNO<br>F(1,23) = 4.993<br><br>CNO ╳ group<br>F(2, 23) = 0.7143
5a, left panel	post-hoc Sidak's test	n = 9 ctrl<br>n = 8 mesoacc<br>n = 9 mesocort	wild-type Crl:WU rats	Ctrl<br>p = 0.6429<br>Mesoaccumbens<br>p = 0.1202<br>Mesocortical<br>p = 0.9186	Ctrl<br>t(23) = 1.082<br>Mesoaccumbens<br>t(23) = 2.156<br>Mesocortical<br>t(23) = 0.5813
5a, right panel	two-way RM anova	n = 9 ctrl<br>n = 8 mesoacc<br>n = 9 mesocort	wild-type Crl:WU rats	CNO<br>p = 0.0096<br><br>CNO ╳ group<br>p = 0.0207	CNO<br>F(1,23) = 7.984<br><br>CNO ╳ group<br>F(2, 23) = 4.612
5a, right panel	post-hoc Sidak's test	n = 9 ctrl<br>n = 8 mesoacc<br>n = 9 mesocort	wild-type Crl:WU rats	Ctrl<br>p = 0.9302<br>Mesoaccumbens<br>p = 0.0017<br>Mesocortical<br>p = 0.9957	Ctrl<br>t(23) = 0.5490<br>Mesoaccumbens<br>t(23) = 3.995<br>Mesocortical<br>t(23) = 0.2082

Fig	Test used	*n*	what?	p value	test statistic
5b, left panel	two-way RM anova	n = 12 mesoacc<br>n = 11 mesocort	wild-type Crl:WU rats	MESOACCUMBENS<br><br>Prefeeding:<br>p < 0.0001<br>Main effect of CNO:<br>p = 0.7745<br>Prefeeding × CNO interaction:<br>p = 0.8448<br><br>MESOCORTICAL<br><br>Main effect of prefeeding:<br>p < 0.0001<br>Main effect of CNO:<br>p = 0.9516<br>Prefeeding × CNO interaction:<br>p = 0.5318	MESOACCUMBENS<br><br>Prefeeding: F(1, 11) = 48.89<br>Main effect of CNO: F(1, 11) = 0.0863<br>Prefeeding × CNO interaction: F(1, 11) = 0.0402<br><br>MESOCORTICAL<br><br>Main effect of prefeeding: F(1, 10) = 58.47<br>Main effect of CNO: F(1, 10) = 0.0039<br>Prefeeding × CNO interaction: F(1, 10) = 0.4195
5b, right panel	two-way RM anova	n = 12 mesoacc<br>n = 11 mesocort	wild-type Crl:WU rats	MESOACCUMBENS<br><br>Main effect of prefeeding:<br>p < 0.0001<br>Main effect of CNO:<br>p = 0.1472<br>Prefeeding × CNO interaction:<br>p = 0.5287<br><br>MESOCORTICAL<br><br>Main effect of prefeeding:<br>p < 0.0001<br>Main effect of CNO:<br>p = 0.4654<br>Prefeeding × CNO interaction:<br>p = 0.8877	MESOACCUMBENS<br><br>Main effect of prefeeding: F(1, 11) = 109.0<br>Main effect of CNO: F(1, 11) = 2.432<br>Prefeeding × CNO interaction: F(1, 11) = 0.4233<br><br>MESOCORTICAL<br><br>Main effect of prefeeding: F(1, 10) = 199.2<br>Main effect of CNO: F(1, 10) = 0.5761<br>Prefeeding × CNO interaction: F(1, 10) = 0.0210
5c, left panel	two-way RM anova	n = 9 ctrl<br>n = 8 mesoacc<br>n = 9 mesocort	wild-type Crl:WU rats	CNO<br>p = 0.0006<br><br>CNO × group<br>p = 0.0007	CNO<br>F(1,23) = 15.58<br><br>CNO × group<br>F(2, 23) = 10.04
5c, left panel	post-hoc Sidak's test	n = 9 ctrl<br>n = 8 mesoacc<br>n = 9 mesocort	wild-type Crl:WU rats	Ctrl<br>p = 0.8998<br>Mesoaccumbens<br>p < 0.0001<br>Mesocortical<br>p = 0.9947	Ctrl<br>t(23) = 0.6289<br>Mesoaccumbens<br>t(23) = 5.776<br>Mesocortical<br>t(23) = 0.2229
5c, right panel	two-way RM anova	n = 9 ctrl<br>n = 8 mesoacc<br>n = 9 mesocort	wild-type Crl:WU rats	CNO<br>p = 0.0204<br><br>CNO × group<br>p = 0.0680	CNO<br>F(1,23) = 6.204<br><br>CNO × group<br>F(2, 23) = 3.029
5c, right panel	post-hoc Sidak's test	n = 9 ctrl<br>n = 8 mesoacc<br>n = 9 mesocort	wild-type Crl:WU rats	Ctrl<br>p = 0.9840<br>Mesoaccumbens<br>p = 0.0082<br>Mesocortical<br>p = 0.9392	Ctrl<br>t(23) = 0.3250<br>Mesoaccumbens<br>t(23) = 3.353<br>Mesocortical<br>t(23) = 0.5219
6b<br>ctrl	Sidak's test<br>(9 comparisons)	n = 8	wild-type Crl:WU rats in ctrl group	no pun vs pun sal<br>p < 0.0001<br><br>no pun vs pun cno<br>p = 0.0002<br><br>pun sal vs pun cno<br>p = 0.9632	no pun vs pun sal<br>t(50) = 4.823<br><br>no pun vs pun cno<br>t(50) = 4.595<br><br>pun sal vs pun cno<br>t(50) = 0.2279

Fig	Test used	*n*	what?	p value	test statistic
6b<br>mesoacc	Sidak's test<br>(9 comparisons)	n = 9	wild-type Crl:WU rats in mesoacc group	no pun vs pun sal<br>p = 0.0002<br><br>no pun vs pun cno<br>p = 0.9995<br><br>pun sal vs pun cno<br>p = 0.0001	no pun vs pun sal<br>t(50) = 4.393<br><br>no pun vs pun cno<br>t(50) = 0.1013<br><br>pun sal vs pun cno<br>t(50) = 4.494
6b<br>mesocort	Sidak's test<br>(9 comparisons)	n = 9	wild-type Crl:WU rats in mesocort group	no pun vs pun sal<br>p < 0.0001<br><br>no pun vs pun cno<br>p < 0.0001<br><br>pun sal vs pun cno<br>p = 0.9942	no pun vs pun sal<br>t(50) = 4.840<br><br>no pun vs pun cno<br>t(50) = 4.407<br><br>pun sal vs pun cno<br>t(50) = 0.4323
6b	2-way anova	n = 26	wild-type Crl:WU rats (all groups combined)	group<br>p = 0.2567<br><br>treatment<br>p < 0.0001<br><br>treatment × group<br>p = 0.0048	group<br>F(2,25) = 4.268<br><br>treatment<br>F(2,50) = 33.59<br><br>treatment × group<br>F(4,50) = 4.268
6c	one-sample t-test	n = 9	TH::Cre rats	p = 0.0074	t(8) = 3.560
6d	two-way RM ANOVA	ctrl<br>n = 8<br><br>mesoacc<br>n = 9<br><br>mesocort<br>n = 9	wild-type Crl:WU rats	CNO<br>p = 0.7490<br><br>CNO × group<br>p = 0.9892	CNO<br>F(1, 23) = 0.1048<br><br>CNO × group<br>F(2, 23) = 0.0109
7a, win trials panel	two-way RM ANOVA	n = 4	TH::Cre rats	CNO<br>p = 0.9483<br><br>CNO × time<br>p > 0.9999	CNO<br>F(1, 3) = 0.00496<br><br>CNO × time<br>F(2000,6000) = 0.3933
7a, lose trials panel	two-way RM ANOVA	n = 5	TH::Cre rats	CNO<br>p = 0.8928<br><br>CNO × time<br>p > 0.9999	CNO<br>F(1, 3) = 0.0215<br><br>CNO × time<br>F(2000,6000) = 0.3843

Supplementary references

1. G. Paxinos and C. Watson. The Rat Brain in Stereotactic Coordinates (6th Edition). Elsevier Inc. (2007).
