# Supplementary material for: A neuronal mechanism underlying decision-making deficits during hyperdopaminergic states
Source: Nat Commun. 2018 Feb 21;9:731. doi: 10.1038/s41467-018-03087-1 (PMC5821846; doi:10.1038/s41467-018-03087-1)
Supplement: Supplementary file 3 — Description of Additional Supplementary Files [file 41467_2018_3087_MOESM3_ESM.pdf]

Description of Additional Supplementary Files

File Name: Supplementary Movie 1
Description: *In vivo* neuronal population activity from DA neurons in the VTA of a TH::Cre rat during reversal learning, measured using fiber photometry.
